# Creating Consensus: Revisiting the Emergency Medicine Resident Scholarly Activity Requirement

**DOI:** 10.5811/westjem.2018.10.39293

**Published:** 2018-12-05

**Authors:** Bryan G. Kane, Vicken Y. Totten, Chadd K. Kraus, Michael Allswede, Deborah B. Diercks, Nidhi Garg, Louis Ling, Eric N. McDonald, Alex M. Rosenau, Mike Wilk, Alexandria D. Holmes, Adam Hemminger, Marna Rayl Greenberg

**Affiliations:** * Lehigh Valley Hospital, Department of Emergency Medicine and Hospital Medicine, Allentown, Pennsylvania; † University of South Florida, Morsani College of Medicine, Lehigh Valley Campus, Allentown, Pennsylvania; ‡ Kaweah Delta Medical Center, Department of Emergency Medicine, Visalia, California; § Geisinger Health System, Department of Emergency Medicine, Danville, Pennsylvania; ¶ Mountainview Hospital, Department of Emergency Medicine, Las Vegas, Nevada; || University of Texas Southwestern Medical Center, Department of Emergency Medicine, Dallas, Texas; # Long Island Jewish Medical Center, Northwell Health, Department of Emergency Medicine, New Hyde Park, New York; ** Accreditation Council for Graduate Medical Education, Chicago, Illinois; †† University of Minnesota, Department of Emergency Medicine, Minneapolis, Minnesota; ‡‡ University of Mississippi, Department of Emergency Medicine, Oxford, Mississippi; §§ Brown University, Department of Emergency Medicine, Providence, Rhode Island

## Abstract

**Introduction:**

In the context of the upcoming single accreditation system for graduate medical education resulting from an agreement between the Accreditation Council for Graduate Medical Education (ACGME), American Osteopathic Association and American Association of Colleges of Osteopathic Medicine, we saw the opportunity for charting a new course for emergency medicine (EM) scholarly activity (SA). Our goal was to engage relevant stakeholders to produce a consensus document.

**Methods:**

Consensus building focused on the goals, definition, and endpoints of SA. Representatives from stakeholder organizations were asked to help develop a survey regarding the SA requirement. The survey was then distributed to those with vested interests. We used the preliminary data to find areas of concordance and discordance and presented them at a consensus-building session. Outcomes were then re-ranked.

**Results:**

By consensus, the primary role(s) of SA should be the following: 1) instruct residents in the process of scientific inquiry; 2) expose them to the mechanics of research; 3) teach them lifelong skills, including search strategies and critical appraisal; and 4) teach them how to formulate a question, search for the answer, and evaluate its strength. To meet these goals, the activity should have the general elements of hypothesis generation, data collection and analytical thinking, and interpretation of results. We also determined consensus on the endpoints, and acceptable documentation of the outcome.

**Conclusion:**

This consensus document may serve as a best-practices guideline for EM residency programs by delineating the goals, definitions, and endpoints for EM residents’ SA. However, each residency program must evaluate its available scholarly activity resources and individually implement requirements by balancing the ACGME Review Committee for Emergency Medicine requirements with their own circumstances.

## INTRODUCTION

### Background

**Figure 1:**
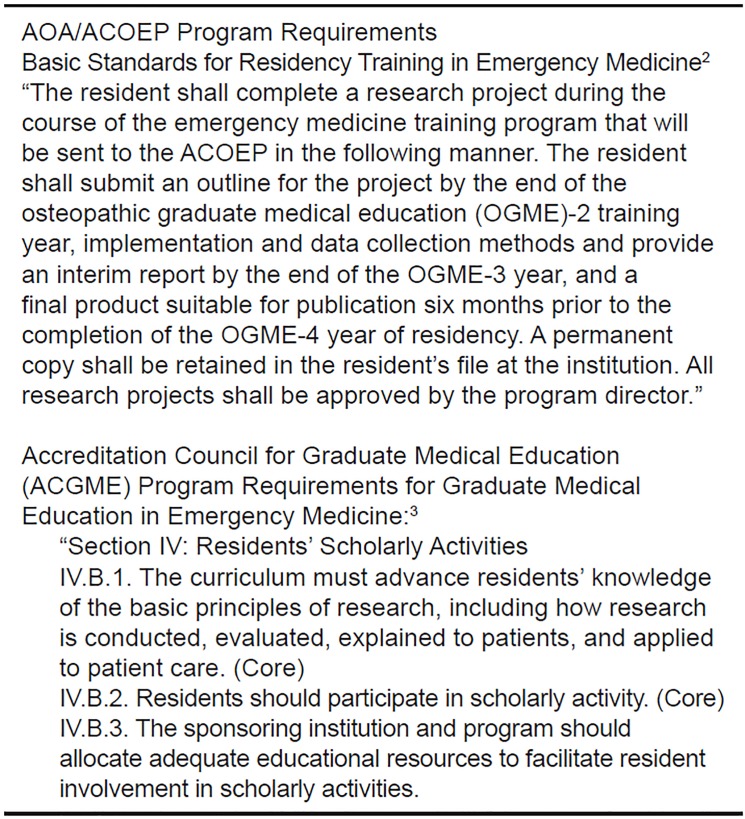
American Osteopathic Association (AOA) and Accreditation Council for Graduate Medical Education (ACGME) emergency medicine resident scholarly activity requirement.

In 1999 the Research Directors’ Interest Group (RDIG) of the Society of Academic Emergency Medicine (SAEM) developed a consensus statement on the emergency medicine (EM) scholarly project requirement for residents.^1^ Program requirements for both American Osteopathic Association (AOA)/American College of Osteopathic Emergency Physicians (ACOEP) and the Accreditation Council for Graduate Medical Education (ACGME) EM residencies identify scholarly activity (SA) as a core requirement of training (Figure 1).^2^,^3^ Additionally, residents in osteopathic programs have a requirement to produce a research project of publishable quality and submit it for review and approval to the ACOEP six months prior to residency graduation.

#### Importance

The initial intent of the SA requirement in the evolution of EM was in part to counter critics who argued that EM did not have robust, specialty-specific literature or the necessary academic productivity to be a distinct specialty.^4^ Therefore, the SA requirement was pressed into service to identify that scope of practice and create that body of evidence. Nearly 40 years after the formal recognition of EM, the need for SA remains, although there is no single approach to it among residencies.^5^ The single accreditation system (SAS) for graduate medical education scheduled to be in place in July 2020, is an unprecedented opportunity for creating a consensus understanding and implementation of a revised SA requirement.

**Population Health Research Capsule**

What do we already know about this issue?

There has been no single approach among residencies for the emergency medicine resident scholarly activity (EM SA) requirement.

What was the research question?

We set out to produce a consensus document on best practices, processes and outcomes for the EM SA.

What was the major finding of the study?

The EM SA should instruct residents in scientific inquiry, expose them to the mechanics of research, and teach them how to formulate a question, search for the answer and evaluate its strength.

How does this improve population health?

Consensus on the endpoints and documentation of the outcome of the EM SA may serve as a best-practices guideline for EM residency programs.

#### Goals

We set out to produce a revised consensus document on best practices, processes, and outcomes for EM SA by engaging relevant stakeholders in a consensus workshop convened by the RDIG and the Evidence-based Healthcare Implementation (EBHI) interest groups of SAEM.

## METHODS

### Study Design

**Figure 2:**
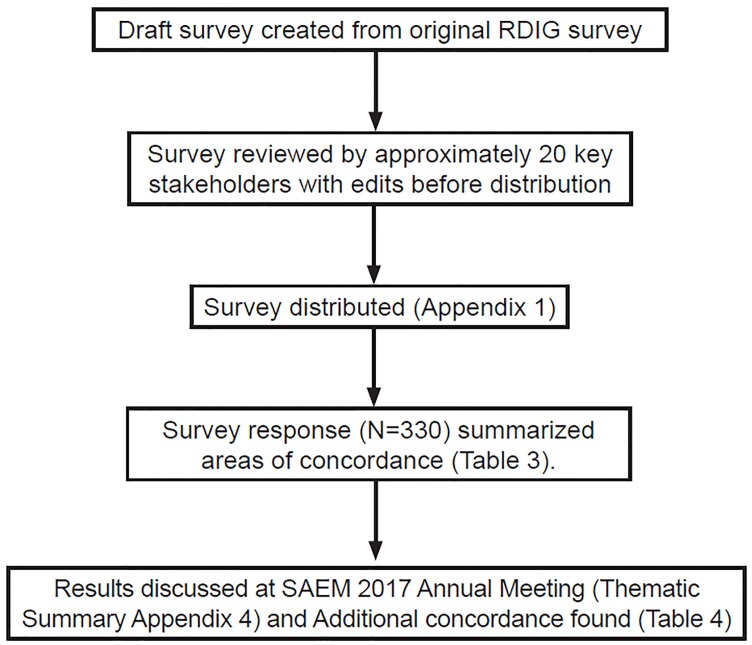
Flowchart of the process in developing consensus on scholarly activity requirements. *RDIG*, Research Directors’ Interest Group; *SAEM*, Society of Academic Emergency Medicine.

The 2017 RDIG and EBHI workshop used similar consensus methodology with a reiterative process of collecting and consolidating ideas from a group of relevant stakeholders in a four-step, consensus-building process (nominal group technique) that was previously used in an *Academic Emergency Medicine* (*AEM*) consensus meeting.^6^ And as with the prior RDIG consensus (1999), this methodology included convening at the annual SAEM meeting.^1^ The institutional review board at the lead author’s institution deferred review to the SAEM board, which reviewed and approved the project. In the months leading up to the consensus meeting, RDIG/EBHI members reviewed prior work on this topic.^1^,^5^,^7^ Based on this research, (flow diagram, Figure 2) a survey was drafted by representatives of interest group membership.

The draft survey included demographic questions about respondents and ranking-scale responses to queries about the goals, definition, and endpoints for the SA as well as the role of the research director in the process. This was largely based on the questions used in the original RDIG survey.^1^ To establish face and content validity, we piloted the survey among approximately 20 expert EM faculty (from diverse geographical regions) involved in resident education and familiar with SA curriculum development and delivery. The key stakeholders were from the following groups: Association of Academic Chairs in EM, Residency Review Committee/ACGME, program directors (PD), and Emergency Medicine Residents’ Association (EMRA). We revised the survey based on feedback from the pilot survey. Revisions were made based on the ACGME focus on quality improvement (QI) on Clinical Learning Environment Reviews visits, and information about knowledge translation/QI were added. After this, and to involve additional expert judgment to support the content validity and to demonstrate that the content would be understood, the survey and project goals were reviewed and approved by the SAEM board without further changes.

### Selection of Participants

We then distributed the survey (Appendix 1) via email to multiple groups with stakeholder interest, including several SAEM interest groups, committees of the American College of Emergency Physicians (ACEP), American Academy of Emergency Medicine (AAEM), and ACOEP, EMRA and AAEM’s resident association (AAEM/RSA), and to PDs and associate/assistant PDs and other EM educators via the Council of Emergency Medicine Residency Directors (CORD) listserv. Instructions to recipient groups were to forward the survey link liberally to any groups or individuals that might have a vested interest in this topic. Participants may have received multiple surveys based on overlapping memberships.

### Intervention, Measurement and Outcomes

The second step of the process included analyzing the results from the returned surveys. Responses were on a four-point Likert scale (from 1=disagree 1, 2=somewhat disagree, 3=somewhat agree to 4=agree). Consensus was defined *a priori* as a ranking of 3.33 or higher. These results were used to find areas of concordance and discordance among the group and were presented at a combined RDIG/EBHI two-hour, consensus-building session at the SAEM annual meeting in May 2017. Stakeholder representatives, who had access to the data in advance, were given the opportunity to briefly present their viewpoints. A few of these presenters were delegates provided by their organizations (ACOEP, EMRA), while others were selected to present due to their availability. A robust group discussion followed.

The third step was re-ranking the outcomes using an anonymous electronic polling system at the interest group meeting. For those who could not access the electronic polling system, a paper form of the poll was available (also submitted anonymously unless respondents elected to identify themselves). Following the group meeting, the results in an abbreviated form were also presented and discussed as a part of a didactic about SA best practices at the SAEM scientific assembly. The fourth step, conducted after the conference, was the summary by the workgroup and included qualitative summarization of the discussion.

### Analyses

Results for demographic variables were reported in simple frequencies and percentages. We reported Likert-ranked results in mean scores.

## RESULTS

### First Iteration

A convenience sample of 330 stakeholders responded to the distributed survey (Appendix 1). Those who agreed to be identified for their participation are listed in Appendix 2. Of the 330 respondents, 54% were affiliated with an EM post-graduate year (PGY) 1–3 program, 44% with a PGY 1–4 program, and 2% other (e.g., family practice (FP)/EM program). The most common age range selected was 31–40 years old; 60% of respondents were male. Organizational representation of participants and their positions can be found in Tables 1 and 2, respectively. Based on our definition of consensus, the primary role of SA, the definition of SA, the minimal endpoints consistent with the definition of the SA, the role of the research director and other respondent findings can be found in Table 3.

The primary focus of the conversation at the combined interest group meeting of the EBHI and the RDIG at SAEM 2017 included describing the elements of minimum standards for a scholarly project, since consensus had not been reached for these elements in the first iteration of the process. In this second iteration, the verbiage “minimal endpoint” was interchanged with “outcome” for SA to meet with the group’s desire to see that the successful completion of the SA requirement should result in the resident submitting to the residency program a measurable product. This product is the outcome of the SA. Therefore, the engagement and discussion at the meeting set out to further clarify what constitutes best-practice, measurable outcomes for the SA.

### Second Iteration

Over 50 participants gathered at the annual SAEM meeting to discuss the resident scholarly project and the data from the survey. Those who agreed to be identified for their participation are listed in Appendix 3. The positions of stakeholder representatives are summarized in Appendix 4. Following these stakeholder position presentations, there was a discussion on content themes as summarized in Appendix 5. After the discussion another iteration of consensus building occurred, facilitated by electronic polling. The group additionally agreed on best-practice, measurable outcomes of the SA. (Table 4)

A summary of the best-practice consensus on the SA has been formatted in a PD handout format (Appendix 6). After the consensus manuscript was prepared, it was reviewed and approved by the board of each of the three major entities in our specialty – SAEM, ACEP, and the ACOEP.

## DISCUSSION

While conceptually some attitudes toward SA have remained the same as in the 1999 RDIG consensus statement on this topic, others have evolved with time. The primary goal for the SA, which is to instruct residents in the process of scientific inquiry, remains a priority. However, four of the previous goals^1^ no longer had the highest ranking of importance (to teach problem-solving skills; to learn the art of medical writing; to expose the resident to research for consideration of an academic career; and to help focus the resident on an area of interest or expertise). Therefore, these four goals have been removed from our current consensus proceedings. In contrast, in respect to the definition of the scholarly project, *all* of the elements of the SA activity identified in the 1999 consensus remained prioritized, 1 along with one additional element – being able to critically appraise the literature.

With regard to the submitted SA outcomes, several proposals did not meet the bar for best practice as determined by this consensus group. Items such as “writing a case report,” “developing a curriculum,” “being a listed member on a consensus policy statement,” “writing and presenting a lecture,” “publishing original research prior to residency,” “participating in or creating an online blog or podcast” all had merit to some participants but did not rank high enough to be considered universally accepted as endpoints. This does not mean that a PD cannot accept any or all of these as acceptable endpoints for either a particular resident or at a particular program. It simply means that these proposals did not rank with the highest concordance of best practice within this group of stakeholders.

Traditional methods to demonstrate SA, such as authorship on peer-reviewed original research publications, will always be one of a number of ways to evaluate scholarly productivity. However, it is also critical to address how to evaluate contributions via non-traditional formats and work products, such as blogs, contributions to FOAMed websites, tweets, etc, which have become the new traditional.^8^ Our findings with regard to the definition of SA may be perceived as more narrow than the more expanded definitions of scholarship and perspectives and discussions on this topic that have shown up in the literature more recently.^9^–^11^ Specifically, these vary from Boyer’s expanded definition of scholarship, which asserts that scholarship should have four separate yet overlapping meanings: the scholarship of discovery; the scholarship of integration; the scholarship of application; and the scholarship of teaching.^9^

It will be incumbent upon stakeholders in the future to address how to measure and recognize these new SA and academic accomplishments and how to create an academic currency from them that can be recognized institutionally (e.g., by university tenure and promotion committees) and externally (e.g., by funding agencies). Innovative metrics are already evolving. For example, altmetrics^12^ helps researchers track and demonstrate the reach and influence of their work beyond traditional citations in peer-reviewed publications. These and other new metrics will impact the ways by which the strength of scholarly effort is measured. Furthermore, while traditionally the research director has had the role of supervising these activities, as non research-based scholarship becomes more prevalent, programs will be needed to determine the most qualified individual(s) to teach and evaluate these efforts.

The work product or output of the SA should imbue lifelong learning skills to the participating resident, with the goal to expand the evidence-based practice of EM and advance the care of patients in the emergency department. EM residents should be in a position to accelerate both knowledge translation and knowledge application. Faculty in EM residency programs should demonstrate academic development that promotes career progression and recognizes competence as mentors and educators preparing residents for academic, administrative, and clinical careers. Departments of EM should benefit from these activities by institutional and extramural recognition. Finally, this residency-training requirement may contribute to the inspiration for a subset of residents to pursue a career in academic EM, thus augmenting this portion of the EM workforce.

SA requirements during residency training should be aimed at equipping residents with skills that take them beyond being mere consumers and implementers of evidence-based medicine to being physicians who can implement the skills learned from SA to continue to develop new knowledge and further the specialty. Additionally, the SA should contribute to faculty and departmental development in a synergistic fashion. Knowledge translation from the time of establishing evidence to the time of adoption into practice traditionally has been delayed by years. At this juncture, the ACGME is in the process of revising the Common Program Requirements. Optimally those changes will both continue to require rigorous scholarship and support the resources (faculty and institutional) to enable the consensus model we have drafted. 13 Additionally, with the SAS on the horizon, there is the opportunity to reshape and redefine the scholarly requirement to better serve patients, trainees, physicians, and the specialty of EM.

## LIMITATIONS

The consensus process has several limitations that were discussed by Summers et al.^1^ and need to be considered when interpreting the results. There is the potential for bias if the representatives of the respective stakeholder organizations expressed their personal opinions rather than the perspective of the organization. However, this was minimized by formally requesting organizations to send representatives. Additionally, it is possible that the individuals who participated in the process do not represent the range of opinions of their organizations on SA. Furthermore, in contrast to experts who usually participate in a nominal group-technique consensus building, a percentage of the consensus participants (residents) were novice learners. Our rationale for the inclusion of residents is that they were clearly vested stakeholders who have expertise in many of the non-research SA areas. It is notable that while they may not be in a position to adequately evaluate how the SA applies to the attending-level, independent practice of EM, we did not collect information to identify how knowledgeable they were in non research-based scholarship.

The total number of survey recipients is not known nor was there available to us a response rate overall for the different stakeholder groups. We have no way of knowing whether those on a listserve actually received the survey. This uncertainty combined with the fact that respondents frequently were members of multiple stakeholder groups made it impossible to dissect these results by group. Furthermore, we were unable to show how many total residency programs were represented and what fraction of all residencies (ACGME and AOA) were represented. Despite these limitations, we feel confident in reporting the initial survey responses because many of the experts identified their perspectives by name, and the ratings of those reported as consensus were consistent. The elements of the survey response that lacked agreement were reviewed and underwent a re-vote in the second iteration of the process. As a consensus project, the response rate and sampling were not as rigorous as one might find in a research study.

## CONCLUSION

Having been approved by the boards of SAEM, ACEP, and ACOEP, this consensus document may serve as a best-practice guideline for residency programs by delineating the goals, definition and endpoints for EM resident scholarly activity. In applying this guiding document, residency programs should evaluate the resources they have available and implement their individual site requirements by balancing the Review Committee in Emergency Medicine requirements with their own circumstances. Future discussion to determine how non-traditional work products can best be evaluated and incorporated into this activity requirement should be encouraged.

## Supplementary Information













## Figures and Tables

**Table 1:** Demographics of organizations represented. (Respondents could check all categories that applied to them.)

Answer choices	Respondents N=321	
SAEM Research Directors’ Interest Group	11.53%	37
SAEM Evidence-based Healthcare Implementation Interest Group	8.41%	27
SAEM Research Committee	10.59%	34
ACEP Research Committee	7.48%	24
ACEP	79.75%	256
SAEM	34.27%	110
AAEM	32.40%	104
ACOEP	26.17%	84
CORD	37.69%	121
EMRA	37.38%	120
AACEM	1.87%	6
ACGME/RRC	4.98%	16
Other (please specify)	7.79%	25

*SAEM,* Society of Academic Emergency Medicine; *ACEP*, American College of Emergency Physicians, *AAEM*, American Academy of Emergency Medicine; *ACOEP*, American College of Osteopathic Emergency Physicians; *CORD*, Council of Emergency Medicine Residency Directors; *EMRA*, Emergency Medicine Residents’ Association; *AACEM*, Association of Academic Chairs of Emergency Medicine; *ACGME*, Accreditation Council for Graduate Medical Education; *RRC*, Residency Review Committee.

**Table 2:** Demographics—positions held (respondents could check all that applied to them).

Answer choices	Responses	
Faculty of EM residency program	35.09%	113
Program director (or assistant PD)	28.26%	91
Research director (or assistant)	15.22%	49
Fellowship director (or assistant)	3.11%	10
Resident/fellow	34.78%	112
Department chair (or vice)	6.52%	21
ACGME/RRC member	1.24%	4
EM physician	21.12%	68
Program coordinator	0.31%	1
Other (please specify)	5.59%	18
Total respondents: 322		

*EM,* emergency medicine; *ACGME*, Accreditation Council for Graduate Medical Education; *RRC*, Residency Review Committee; *PD*, program director.

**Table 3:** First iteration.

Category	Ranking
Primary role of the scholarly activity	
Instruct the resident in the process of scientific inquiry	3.48
Expose the resident to the mechanics of research	3.51
Teach the resident lifelong skills including search strategies and critical appraisal	3.38
Teach the resident how to formulate a question, search for the answer, and evaluate the strength of the answer	3.41
Definition of the scholarly activity	
Should include the general elements of hypothesis generation	3.53
Information gathering or data collection	3.61
Evidence of data analysis or analytical thinking	3.47
Interpretation of results or statement of conclusion	3.6
Being able to critically appraise medical literature	3.52
Role of the research director	
Help set the guidelines for the scholarly activity	3.51
Check timeline for project completion	3.37
Help create a departmental environment for research	3.74
Help provide tools and resources for research	3.8
Act as a motivator for scholarly activity among residents	3.67
Instruct the resident in critical appraisal skills	3.63
Endpoints consistent with the definition of the scholarly project	
A public health project	3.62
A quality improvement exercise	3.47
A systematic review	3.54
A paper of publishable quality	3.81
A published, original research paper	3.92
Developing an evidence-based practice guideline	3.47
A book chapter	3.45
Other	
The activity can be spread over three or more years	3.39
Responsibility of the project primarily rests with the resident	3.66
Responsibility of the project is supported by a combination of the resident, the program and research directors.	3.37

**Table 4:** Outcome of the scholarly activity requirement for residents in emergency medicine.

Category	Ranking
Outcome of the scholarly activity	
Written documentation of the project archived by the residency	3.73
A developed and implemented protocol (research or quality improvement)	3.80
A research paper that includes a hypothesis, collected and analyzed data (or showed analytical thinking), and a conclusion (or interpretation of results)	3.93
A research abstract submission	3.55
An oral research presentation	3.61
