# Supplementary material for: Creating Consensus: Revisiting the Emergency Medicine Resident Scholarly Activity Requirement
Source: West J Emerg Med. 2018 Dec 5;20(2):369–75. doi: 10.5811/westjem.2018.10.39293 (PMC6404691; doi:10.5811/westjem.2018.10.39293)
Supplement: Supplementary file 1 [file wjem-20-369-s001.pdf]

Please help us by completing this brief survey by April 9, 2017. You are receiving it because you were identified as someone who may be interested in helping us build consensus on the 'scholarly project' requirement for EM residents. It has been 18 years since the SAEM Research Directors Interest group drafted a consensus on this topic and we are revisiting it in collaboration with the Evidence Based Healthcare and Implementation Interest Group. The responses you submit will be reported in an aggregate deidentified format at the SAEM 'Scholarly Project' Workshop which will be held during the combined interest group meetings at SAEM. While it will be helpful, it is not mandatory that you be at the SAEM meeting. If you are able, please join us in Orlando on :

Thursday, May 18, 2017
7:00 AM - 8:50 AM
Bayhill 19: Lobby Level

Thank you in advance for your contribution.

Bryan Kane, MD
Chair, EBHI and
Marna Greenberg, DO, MPH
Chair, RDIG

1. Purpose

On a scale of 1 to 4 (1 not very important to 4, very important) a primary role of the scholarly project is to:

	1	2	3	4
Instruct residents in the process of scientific inquiry	○	○	○	○
Teach the resident problem-solving skills	○	○	○	○
Expose the resident to the mechanics of research	○	○	○	○
Learn the art of medical writing	○	○	○	○
Expose the resident to research for consideration of an academic career	○	○	○	○
Help focus the resident on an area of interest or expertise (EMS, toxicology, critical care, etc.)	○	○	○	○
Teach the resident lifelong skills, including search strategies and critical appraisal	○	○	○	○
Teach the resident how to apply best practice principles to improve the delivery of health care in their system	○	○	○	○
Teach the resident how to formulate a question, search for the answer, and evaluate the strength of the answer	○	○	○	○

Other, please describe:

2. Definition.

On a scale of 1 to 4 (1 not very important, 4 very important), at completion of the scholarly project a resident should know how to:

	1	2	3	4
Generate a hypothesis	◯	◯	◯	◯
Gather information and/or collect data	◯	◯	◯	◯
Analyze data or show evidence of analytical thinking	◯	◯	◯	◯
State a conclusion or interpret results	◯	◯	◯	◯
Contribute to what is known	◯	◯	◯	◯
Have the potential to change the health care system	◯	◯	◯	◯

Other, please describe:

3. Endpoints.

On a scale of 1 to 4 (1 not very important, 4 very important), the scholarly project should at minimum result in:

	1	2	3	4
A research proposal submitted to the IRB	◯	◯	◯	◯
An abstract submission	◯	◯	◯	◯
An oral presentation	◯	◯	◯	◯
A paper of publishable quality that includes all of these elements: hypothesis, data collection, analysis, interpretation	◯	◯	◯	◯
A paper submitted to a peer-reviewed journal	◯	◯	◯	◯
Written documentation of the project archived by the residency	◯	◯	◯	◯

Other, please describe:

4. Endpoints (cont.)

On a scale of 1 to 4 (1 completely disagree, 4 completely agree):

	1	2	3	4
A case report can meet the definition of the scholarly project.	○	○	○	○
A public health project can meet the definition of the scholarly project.	○	○	○	○
A quality improvement exercise can meet the definition of the scholarly project.	○	○	○	○
A curriculum development can meet the definition of the scholarly project.	○	○	○	○
A lecture can meet the definition of the scholarly project.	○	○	○	○
A systematic review can meet the definition of the scholarly project.	○	○	○	○
A published original research paper can meet the definition of the scholarly project.	○	○	○	○
A published original research paper completed prior to starting residency can meet the definition of the scholarly project.	○	○	○	○
Developing an evidence based practice guideline can meet the definition of the scholarly project.	○	○	○	○
A book chapter can meet the definition of the scholarly project.	○	○	○	○
A contribution to an online EM blog or podcast can meet the definition of the scholarly project .	○	○	○	○

Other, please describe:

5. Other.

Regarding implementation of the scholarly project, on a scale of 1 to 4 (1 completely disagree, 4 completely agree), the scholarly project should be:

	1	2	3	4
Completed during one ‘research’ month	◯	◯	◯	◯
Spread out over 3 or more years	◯	◯	◯	◯
The beginning of a career focus	◯	◯	◯	◯

Other, please describe:

6. On a scale of 1 to 4 (1 completely disagree, 4 completely agree) the research director should:

	1	2	3	4
Help set the guidelines for the scholarly activity	◯	◯	◯	◯
Using FINER criteria, provide quality assurance in project development	◯	◯	◯	◯
Check timeline for project completion	◯	◯	◯	◯
Help create a departmental environment for research	◯	◯	◯	◯
Help provide tools and resources for research	◯	◯	◯	◯
Act as a motivator for scholarly activity among residents	◯	◯	◯	◯
Instruct the resident in critical appraisal skills	◯	◯	◯	◯
Instruct the resident in research skills	◯	◯	◯	◯

Other, please describe:

7. On a scale of 1 to 4 (1 completely disagree, 4 completely agree) the person responsible for the resident scholarly project should be:

	1	2	3	4
The resident.	◯	◯	◯	◯
The program director.	◯	◯	◯	◯
The research director.	◯	◯	◯	◯
The resident, program director and the research director.	◯	◯	◯	◯

Other, please describe:

Demographics

8. What is your age?

- ◯ 18-24
- ◯ 25-30
- ◯ 31-40
- ◯ 41-50
- ◯ >51

9. What is your sex?

- ◯ Male
- ◯ Female
- ◯ Other (please specify)

10. Category that best represents your position (mark all that apply):

- ☐ Faculty of EM Residency Program
- ☐ Program Director (or Assistant PD)
- ☐ Research Director (Or assistant)
- ☐ Fellowship Director (or Assistant)
- ☐ Resident/Fellow
- ☐ Department Chair (or Vice)
- ☐ ACGME/RRC member
- ☐ EM physician
- ☐ Program coordinator
- ☐ Other (please specify)

11. What type structure is your program?

- ○ PG 1-3
- ○ PG 1-4
- ○ Other (please specify)

12. Organization that you belong to/represent (mark all that apply):

- ☐ SAEM Research Director's Interest Group
- ☐ SAEM Evidence Based Healthcare Implementation Interest Group
- ☐ SAEM Research Committee
- ☐ ACEP Research Committee
- ☐ ACOEP
- ☐ EMRA
- ☐ AACEM
- ☐ ACGME/RRC
- ☐ Other (please specify)

Thank you for your time!

13. Please provide any additional comments or suggestions:

14. OPTIONAL: The responses you have given will be reported in an aggregate deidentified format. If you want your name and information to be listed generically as a participant in the consensus building process please include it here:

Name: first, last, degree(s)

Institutional Affiliation

Rank and/or Title

email address:
