# Supplementary material for: Creating Consensus: Revisiting the Emergency Medicine Resident Scholarly Activity Requirement
Source: West J Emerg Med. 2018 Dec 5;20(2):369–75. doi: 10.5811/westjem.2018.10.39293 (PMC6404691; doi:10.5811/westjem.2018.10.39293)
Supplement: Supplementary file 2 [file wjem-20-369-s002.docx]

Participants who opted to be Recognized Round 1

| Adler, David, MD MPH |
| --- |
| John Ashurst DO MSc |
| Aaron Barksdale |
| Gillian, Beauchamp, MD |
| Jessica Bennett do |
| Richard Bounds, MD, FACEP |
| Matt Bombard DO |
| Brad Burmeister, MD |
| Elizabeth Burner, MD MPH |
| Richard Byyny, MD, MSc, FACEP |
| Carlos A. Camargo, Jr., MD, DrPH, FACEP |
| Christopher R. Carpenter, MD, MSc, FACEP, AGSF |
| Bharath Chakravarthy MD MPH |
| Sunday Clark, ScD |
| Darren Dillard, DO/MS |
| David Druga, MD |
| Carly Eastin, MD |
| Frank Edwards, MD |
| Barnet Eskin, MD, PhD |
| Abra Fant MD MS |
| Leigh Evans MD |
| Christopher Fee, MD |
| James Feldman MD MPH |
| Caroline Freiermuth MD |
| Jessica Fujimoto MD |
| Nidhi Garg, MD |
| Andrew Goodrich, DO, MS |
| Richard Gordon |
| Stacey House, MD, PhD |
| Bradley Gutierrez, D.O., FACOEP |
| Scott Gutovitz, MD FACEP |
| Joshua Enyart, DO |
| Theodore Gaeta, DO, MPH |
| Michael Gisondi, MD |
| Marna Rayl Greenberg, DO, MPH |
| Kathryn, Hawk, MD, MHS |
| Patrick Hinfey |
| Judd Hollander, MD |
| Mary Hughes DO |
| Aloysius Humbert, MD |
| Derek Hunt, D.O. |
| Natalie Htet, MD, MS |
| Michael Iacono MD |
| Jennifer S. Jackson, MD |
| Jeanne Jacoby MD |
| David Jones, MD MCR |
| Elizabeth Jones, MD |
| Barkataki, Kieron, DO |
| Barry Knapp MD |
| Randy Kring, MD |
| Calvin Krom, D.O. |
| Margaret Mary S. Landel |
| Aaron Lane D.O. |
| Spenser Lang, MD |
| Patrick Lank, MD, MS |
| Cedric Lefebvre, MD |
| David Levy |
| Alexander Limkakeng, MD, MHSC, FACEP |
| Bruce Lo, MD, MBA |
| Seth Lotterman, MD |
| Leann Mainis, MD |
| Dan Mayer, MD |
| Alyson, McGregor, MD |
| Samantha Myers, MD |
| Samuel N.Mark.MD.FACEP |
| Michael Menchine, MD, MPH |
| Joseph Miller, MD, MS |
| Sarah, Mott, MD |
| Robin Naples, MD |
| Steven Nazario, MD |
| Joan Noelker, MD |
| Erik Nordquist, MD |
| Jim Olson |
| James Paxton, MD MBA |
| Ronald G. Pirrallo, MD, MHSA |
| Scott Plasner, DO, FACOEP, FAAEM, FACOI |
| Michael Puskarich |
| Megan Ranney MD MPH |
| Linda Regan |
| Michael Repplinger, MD, PhD |
| Daniel L. Richardson, DO, FACEP |
| Fleischman, Ross, MD, MCR |
| Steven Sattler DO |
| Daniel, Schroder DO |
| Jenna Shenk |
| Richard Sinert |
| Sean P. Stickles, MD |
| Heather T. Streich, MD |
| Matthew Streitz, MD |
| Tania D. Strout, PhD, RN, MS |
| Leonard Stallings, MD |
| Megan Stobart-Gallagher, DO |
| Vicken Totten MD |
| Stacy Trent, MD MPH |
| Muhammad Waseem |
| Kevin Weaver D.O. |
| Grant Wei, MD |
| R. Gentry Wilkerson, MD |
| Allan B Wolfson, MD |
| Kabir Yadav MDCM MS MSHS |
| Michael Yangouyian, D.O. |
