# Supplementary material for: Creating Consensus: Revisiting the Emergency Medicine Resident Scholarly Activity Requirement
Source: West J Emerg Med. 2018 Dec 5;20(2):369–75. doi: 10.5811/westjem.2018.10.39293 (PMC6404691; doi:10.5811/westjem.2018.10.39293)
Supplement: Supplementary file 3 [file wjem-20-369-s003.docx]

**Appendix 3 Participants in Round 2 of the RDIG/EBHI Combined Interest Group Consensus Building Meeting at SAEM, May 18, 2017.**

David H. Adler MD MPH , John R. Allegra MD PhD, Michael Allswede DO, Ryan C. Arnold MD, Aaron Barksdale MD , Lars Beattie MD MS, Michael Brown MD MSc, Jestin N. Carlson MD, John Cienki MD, Sunday Clark ScD. Richelle Cooper MD MSHS, Kathleen Cowling MS DO, Stephen Davis MS, Deborah Diercks MD, Carly Eastin MD, Rakesh Engineer MD,

Barnet Eskin MD, Jennifer Frey PhD, Gary M. Gaddis MD PhD, Nidhi Garg MD

Marna Rayl Greenberg DO MPH, Kari Harland MPH PhD, Steven M. Hochman MD

James F. Holmes, Jr. MD MPH, Peter C. Hou MD, Bryan G. Kane MD, Chadd Kraus DO,

Louis J. Ling MD, S.V. Mahadevan MD, Dan Mayer MD, Eric McDonald, MD

Melissa McMillian CNP, Angela Mills MD, Bryn Mumma MD MAS, Laura Oh MD,

Nicholas Olah, Claire Pearson MD, Ronald G. Pirrallo MD MHSA, Linda Regan MD,

Raymond Regan MD, Michael Repplinger MD PhD, Elissa Schecter-Perkins MD MPH,

Johnathan Sheele, MD MPH, Richard H. Sinert DO, Matthew Strehlow MD, Esteban C. Torres,

Vicken Y. Totten MD MS, Henry Wang MD MS, Muhammad Waseem MD, Michael Wilk MD,

R. Gentry Wilkerson MD, Peter Wyer, MD
