# Supplementary material for: Creating Consensus: Revisiting the Emergency Medicine Resident Scholarly Activity Requirement
Source: West J Emerg Med. 2018 Dec 5;20(2):369–75. doi: 10.5811/westjem.2018.10.39293 (PMC6404691; doi:10.5811/westjem.2018.10.39293)
Supplement: Supplementary file 4 [file wjem-20-369-s004.docx]

Resident Perspective (EMRA and AAEM RSA)

Current ACGME requirements support a diverse definition of qualifying scholarly projects from peer-review publications to non-peer review projects such as podcasts, videos, case reports, textbooks/chapters, conference presentations, and QI research projects.
 Residents continue to support these broad criteria and encourage broadening the definition to mirror the evolving way residents learn and train. One area for expansion is national leadership. Members of the board of directors of national emergency medicine organizations are frequently involved in projects for their respective boards which grow their careers and the specialty.  By current ACGME guidelines, these time consuming activities satisfy the definition of scholarly activity for faculty, but lack clarity concerning resident requirements.
 While the benefits of involvement in research projects to enhance understanding of the process is not argued, residents feel that journal club discussions of published papers provide superior education in research practice and interpretation; exposing residents to a variety of research methodologies and a more comprehensive understanding of EM literature. A narrowed definition of scholarly activity which only includes peer reviewed research would limit resident opportunity, education, and ignore the great work done by residents in non-traditional knowledge translation. The broad definition of scholarly projects should continue to exist to support the diverse and unique interests of each individual emergency medicine resident. Residents continue to support this ACGME definition.

The Program Director’s Perspective

The first iteration of this consensus process had 28 percent of the survey respondents identifying as program directors (or associate/assistant program directors). The Program Director (PD) communities are supportive of the SA, although there is concern about the requirement. PDs specifically think that the SA requirement often results in administrative oversight which is time better dedicated to conducting their own academic projects. The primary PD concern is being held accountable by the ACGME on inspections for ensuring residents complete a SA project in environments where the necessary resources to successfully complete the project are not present. Having options for residents to participate in SA based on the individual resident’s interests and career goals is important to PDs. This would allow specific residents to be engaged in SA based on their personal career goals.

ACOEP Perspective

The ACOEP has always had a research-specific, rather than a broad SA, requirement for residents training in osteopathic EM programs. The rationale has been that by doing a small research project, the resident gains an appreciation and understanding of research methodology. Many osteopathic residency programs are in smaller community or rural hospitals, making available resources for resident research highly variable. The ACOEP only recently has begun to require core teaching faculty to publish. Historically core faculty scholarly activity took the form of lectures and committee membership. This resulted in a limited supply of faculty mentors. Despite resource and mentorship limitations, residents were able to participate in research projects. There may be lessons learned which can help the broader ACGME EM community given the PD concerns already noted. These lessons are vital to both the residency program and individual resident’s success because in order to graduate and be board-eligible, a paper describing the research project must be reviewed and accepted by the ACOEP. Over the past 5 years, the ACOEP Research Committee has evaluated these research projects in a structured, comprehensive manner. Those programs with a robust research infrastructure are at an advantage in producing high quality research projects, and are recognized with a national award. Each resident paper receives external, specific feedback similar to peer review. With the upcoming SAS, merging the different scholarly activity requirements, which are currently more stringent for residents from the ACOEP and more stringent for the faculty under the ACGME, will have an impact on both training and the scholarship of the specialty.

The Chair Perspective

Resident scholarly work can increase visibility of an academic institution and of the faculty that supervise them. In an academic center, the role of the Chair/Chief is to provide adequate support for the residency program to meet the resident SA requirement. This support often includes faculty effort and coordinator time, and may include monetary support. Therefore, the Chair / Chief must ensure alignment of these activities with department priorities and faculty promotion requirements.

The challenge is ensuring that faculty have adequate time to both support resident scholarly work and meet the requirements needed for their own career development. Alignment of both is ideal. For example, high caliber activities resulting in peer reviewed publications, or projects resulting in institution practice changes. Given the varying promotion and tenure requirements across institutions, the Chair / Chief also needs to ensure alignment of SA with these requirements. For example, in an institution that does not recognize book chapters or performance improvement projects as scholarly activity, it may be difficult for faculty to devote time to this sort of resident scholarly work. Aligning institutional goals for promotion with the types of resident scholarly work may provide most solid foundation for success.

The ACGME Perspective

The intent of the RRC-EM SA requirement is to develop the necessary skills to engage in life-long learning and to implement evidence-based patient care.^1^ When a resident does scholarly activity, they should experience and answer important questions such as: What is valid and relevant research? How do you personally learn about this research? How do you disseminate knowledge you develop? How do you put together new ideas in an organized fashion? These questions require more than just a didactic session but the actual practice of skills, through the conduct of a project that they will need for the rest of their career. These skills are even more important than learning mere facts, especially given the pace of biomedical discovery.

The variety of research, critical appraisal, and implementation skills the specialty will need from EM practitioners moving forward is vast. As such, the ACGME website gives examples of many things that qualify. Consider resident SA to include patient safety and quality improvement, as well as traditional research. The minimum requirement is participation so that a resident can learn the concepts; it is not necessary for each resident to have a separate project. Some residents and some programs may do more than the minimum to meet the RRC requirements. Each program must ask “what do my residents need? The RRC-EM feels strongly that each program should meet the requirement in its own way and the best practice for one program might not be the best practice for another program. This consensus process is intended to provide examples for programs of best practices for the SA.

The RDIG Perspective

EM as a field now has measurable research output. Therefore, part of the original rationale of the SA has been fulfilled: to generate a body of academic work within EM. As such, previously defined key steps of generating a question or hypothesis, designing the methods, gathering data, and subsequent analysis and dissemination may need to be re-interpreted or broadened. When considering, for example, a systematic review, the data gathered and analyzed may be the rigorous location and assessment of literature. Expanding the definitions of the SA from the prototypical RCT to other, accepted, peer reviewed output is something to be strongly considered. Part of this consideration will need to include how to acknowledge newer forms of academic credit, including “hits” to a particular article, as an example of the impact of any particular project. Finding creative ways to manage the SA requirement at institutions with limited research support resources is also something to consider. Given the ACOEP requirements being implemented at institutions with austere research environments, these programs may have already implemented innovative ideas. One example, suggested by the RRC-EM above, may be to cohort residents onto a larger project, especially one that can be segmented into smaller sections. Finding ways to align interests may also be an important way to create a culture of SA success, which a key component of the Chair’s perspective. Having residents work on a lineage of research core to a faculty members career tract and promotion is one possible alignment given that the ACGME, and more recently the AOA, has clear and specific requirements for faculty to produce peer reviewed literature, when residents are part of a larger team under the direction of a motivated attending the resident is more likely to achieve success.

The EBHI Perspective

Fundamentally, fewer residents want to be producers of the literature than those who will need to consume it. While involvement in an individual SA project as a resident should positively impact that resident’s appreciation for and understanding of well-designed and executed scientific investigation, this benefit is hypothetical and likely limited to a degree to the methodology used in that specific SA. Finding a way to expand the definition of the SA to include modern critical appraisal skills should provide the resident with an experience which has broader and deeper downstream implications as well as a measureable academic output. The example of how to consider a systematic review from the perspective of the original SA definitions is a clear example. Taken a step further, local appraisal followed by implementation of best practices represents a clear need in modern medicine. The barriers to Knowledge Translation (KT) or Implementation Science have been well described. ^2^ Training EM residents to be leaders in KT is a step towards comparative effectiveness methodology and research, a priority for federal agencies.^3^ While not necessarily at a level as robust as comparative effectiveness is local Quality Improvement (QI). QI often requires significantly less regulatory oversight, addressing a previously noted concern. It can allow for measureable peer-reviewed output. Most importantly, it may receive significant residency administrative support because of the universal resident requirement for involvement in patient safety, and QI is a critical component of the Clinical Learning Environment Review (CLER) visit. With CLER visits mandated by the ACGME every 18-24 months, focusing the SA on rigorous QI, with the intention to publish, will further align interests. Evolving the SA to include critical appraisal and patient safety/QI, may meet the discussed interests of residents and as noted satisfy the RRC-EM. The decreased oversight and potential reduced need for statistical support appears to align with the view of Program Directors, while institutional changes have been noted to be important from the perspective of the Chairs. Working on implementation projects may also expose residents to important administrative skills as part of their SA.

References:

1. Sullivan C, Davis F, Ling L. Scholarly Activity Program Requirements: The Review Committee-Emergency Medicine (RC-EM) Perspective. Acad Emerg Med. 2015;22(11):1345-47.

2. Azimi A, Fattahi R, Asadi-Lari M. Knowledge translation status and barriers. J Med Libr Assoc. 2015 Apr; 103(2):96-99.

3. Methods Guide for Effectiveness and Comparative Effectiveness Review. AHRQ Publication No. 10(14)-EHC063-EF. Rockville, MD: Agency for Healthcare Research and Quality. January 2014. [https://www.effectivehealthcare.ahrq.gov/ehc/products/60/318/CER-Methods-Guide-140109.pdf Accessed 07/19/2017](https://www.effectivehealthcare.ahrq.gov/ehc/products/60/318/CER-Methods-Guide-140109.pdf%20Accessed%2007/19/2017)
