# Supplementary material for: Creating Consensus: Revisiting the Emergency Medicine Resident Scholarly Activity Requirement
Source: West J Emerg Med. 2018 Dec 5;20(2):369–75. doi: 10.5811/westjem.2018.10.39293 (PMC6404691; doi:10.5811/westjem.2018.10.39293)
Supplement: Supplementary file 5 [file wjem-20-369-s005.docx]

**Appendix 5 Thematic Summary of the Consensus Meeting Discussion**

While the meeting achieved consensus in the aforementioned areas, abundant discussion therein introduced four major themes: Goals of the Scholarly Project, Outcomes of the Scholarly Project, Development of Emergency Medicine as a Profession, and External Influences on Scholarly Activity. This summary is to illustrate the diversity of opinion during the process.

**Goals of the Scholarly Project**

*Professional Development***:** The resident scholarly project should aim to produce graduates who are intelligent consumers of medical literature. Personally developing or participating meaningfully in a research project facilitates insight on the research process and allows for better judgment when digesting literature. Attendees agreed that the scholarly project should teach residents to ask questions, digest scientific information, develop methods, and use critical appraisal skills, so that upon entering a career, they have the tools to continue life-long learning. While recognizing the importance of the scholarly project as an avenue of professional growth, it was also acknowledged that said growth is largely dependent on resident interest in the project, and programs should explore a broadened framework for the definition of scholarly work to include more robust options for varied interests. Of note, although competency in research methods is considered a goal of the scholarly project, discussion surrounding publications prior to residency revealed the opinion that such projects are more than likely unrelated to Emergency Medicine and thus cannot count as relevant experiential learning.

*Utilization of Scientific Methods and Evidence-Based Medicine:* Topics in evidence-based medicine and critical appraisal, while often integral to completing a successful scholarly project, are separate entities from the project itself. Evidence-based medicine and research methods outlined in RC/ACGME objectives can be gleaned from entities such as Journal Club or didactic sessions, but the resident scholarly project serves to cement knowledge through application.

*Faculty Development:* Some concerns surrounding requirements for the scholarly project are rooted in meeting RC/ACGME standards, including those set for faculty research. Outcomes are best when interests are aligned and residents collaborate with faculty at their respective institution. With collective efforts, scholarly activity completion will satisfy both the resident requirements and faculty requirements for departmental promotion. It was also noted that given the capability of faculty to work on more longitudinal research, faculty should have greater responsibility to produce meaningful research such as randomized control trials.

**Outcomes of the Scholarly Project**

*A Measurable Result:* The outcome measure and academic pattern discussed as an outcome should be able to sustain the definition. As trainers, we have to set high expectations, and peer-reviewed, printed deliverables are held as most impactful. In the past, the resident scholarly project required output of a manuscript of publishable quality. While discussion recognized this as a past ideology and was perceivably open to exploring options moving away from just the requirement of a manuscript with publishable quality, it was reinforced that residents still must output something meaningful and measurable. How do we create evidence of what residents have accomplished without creation of a peer reviewed publication? It is difficult to measure the impact of a blog. Attendees identified the need a tool kit for residency programs to feel comfortable objectively tracking the outputs of the resident scholarly project. Ultimately, attendees agreed that resident output from the project should demonstrate validity, competence, and publishable quality. One option presented as measurable output was having the resident present the project, even at a local research conference or to the local hospital board.

*A Point System:* Some programs have created a point system so that if residents have the desire to complete “less scholarly” projects including case reports, medical photography, or blogs, they can do so to complete their scholarly requirement so long as all the elements required in the definition are met. Through this concept, such “less scholarly” projects would not carry as many points as a published peer review article, but residents could make up for diminished complexity with increased quantity. This concept also allows for residents to develop their own interests, or niche within Emergency Medicine.

*A Piecewise Approach:* The resident scholarly activity could potentially result in larger, more quality projects if those projects are carved into manageable, tangible parts. A resident can still develop an idea, but research directors can help the resident determine what they will be able to complete in 2-3 years. The remainder of the project will then be passed on to a junior resident in a legacy fashion.

*Lack of Standardization as Distinction, Room for Niche among Programs:* Some attendees questioned whether residency programs should actually seek standardization for scholarly activity output. Residents coming in are consumers and may want to have a choice in selecting a program specific to their own needs and career aspirations. Perhaps they may choose a program that does focus heavily in research, or a program that has great opportunities for mentorship, or prospects for quality improvement processes. The way the resident scholarly project is interpreted may be a feature that helps distinguish programs, so that residents may choose one to best meet their long-term goals and interests.

**Development of Emergency Medicine as a Profession**

*Preserving the Growth of Emergency Medicine:* The resident scholarly project also aims to preserve the growth of Emergency Medicine – both the growth of scientific personnel or clinician scientists, and our domain as a specialty. There is an enormous amount of money spent training residents, and part of their payback is contributing to the advancement of Emergency Medicine. Residency programs have an obligation to generate research that directs Emergency Medicine in the right direction. The amount of literature generated by residency programs ultimately directs how Emergency Medicine is going to progress in the future. By diluting the research requirement, or requiring residents to complete projects they are not passionate about, there is a risk of diluting quality of work produced and thus the advancement of Emergency Medicine.

*Integrity of Research:* In an era where technology is so present in healthcare, is the integrity of knowledge and scientific fact at risk? Many admitted they are cautious to accept scholarly activity involving unofficial publications such as blog posts, because peer reviewed journal articles have traditionally been the way of ensuring dissemination of advances in knowledge are valid and reliable.

**External Factors Influencing Scholarly Activity**

*Perceived Resident Attitudes and Interests:* While there are residents who are research-oriented, many are not interested in clinical research, and are more interested in producing other scholarly works including case reports, blog posts, and other medical writing. Many residents are concerned about the notion of completing a scholarly research project simply to “check a box.” Residents have the most success in completing a meaningful project when they have vested interest. While programs want residents to complete clinical research as part of their scholarly activity, it is important that residents are interested in what they are doing, that we let them explore their niches and direct their career paths. Broadening the desired outcomes of the scholarly project in order to accommodate resident interests would also allow faculty to concentrate on developing and mentoring residents who are truly interested in research. Resident involvement should be tailored in a way that it does not add stress to the resident life

*Program Resources and Limitations*: The scholarly project is highly dependent on program resources, including but not limited to time, faculty commitment, departmental resources, and access to external resources like epidemiologists, NIH funding, and robust facilities. The variety of resources available between smaller and higher-powered institutions creates a heterogeneity to the way things are done, and this should be considered. With the limitations of smaller programs, it is ideal to maximally utilize resources at hand to decrease the tremendous effort required to get every resident involved in meaningful research, while at the same time setting expectations for the scholarly project that are doable at the local level.

*Expert Non-Physician Involvement:* Non-physicians such as epidemiologists may be underutilized in facilitating meaningful scholarly activity. These experts in areas such as epidemiology and biostatistics could be an untapped resource in terms of leading resident scholarly activity and helping residents with research infrastructures such as methodology, handling IRB submissions and fielding IRB responses.
