# Supplementary material for: Creating Consensus: Revisiting the Emergency Medicine Resident Scholarly Activity Requirement
Source: West J Emerg Med. 2018 Dec 5;20(2):369–75. doi: 10.5811/westjem.2018.10.39293 (PMC6404691; doi:10.5811/westjem.2018.10.39293)
Supplement: Supplementary file 6 [file wjem-20-369-s006.docx]

Supplement. Best Practice Consensus on SA (Handout for Program Directors)

| **Primary Role of the Scholarly Activity:** |  |  |
| --- | --- | --- |
| Instruct the resident in the process of scientific inquiry |  |  |
| Expose the resident to the mechanics of research |  |  |
| Teach the resident lifelong skills including search strategies and critical appraisal |  |  |
| Teach the resident how to formulate a question, search for the answer, and evaluate the strength of the answer |  |  |
|  |  |  |
| **Definition of the Scholarly Activity:** |  |  |
|  |  |  |
| Should include the general elements of hypothesis generation |  |  |
| Information gathering or data collection |  |  |
| Evidence of data analysis or analytical thinking |  |  |
| Interpretation of results or statement of conclusion |  |  |
| Being able to critically appraise medical literature |  |  |
|  |  |  |
| **Endpoints/Outcomes Consistent with the Definition of the Scholarly Project:** |  |  |
| A public health project |  |  |
| A quality improvement exercise |  |  |
| A systematic review |  |  |
| A paper of publishable quality |  |  |
| A published original research paper |  |  |
| Developing an evidence based practice guideline |  |  |
| A book chapter |  |  |
| A developed and implemented protocol (research or quality improvement) | | 3.8 |
| A research paper that includes: a hypothesis, collected and analyzed data  (or showed analytical thinking), and a conclusion (or interpretation of results) | | 3.93 |
| A research abstract submission | | 3.55 |
| **Other:**  Written documentation of the project should be archived by the residency | | 3.61 |
| The activity can be spread over three or more years | |  |
| Responsibility of the project primarily rests with the resident | |  |
| Responsibility of the project is supported by a combination of the resident, the program and research directors | |  |

| **Role of the Research Director:** |
| --- |
| Help set the guidelines for the scholarly activity |
| Check timeline for project completion |
| Help create a departmental environment for research |
| Help provide tools and resources for research |
| Act as a motivator for scholarly activity among residents |
| Instruct the resident in critical appraisal skills |
|  |
